# Marital Effects in Later Life: Dyadic Approaches and Gender Differences

**DOI:** 10.1093/geroni/igaa057.2040

**Published:** 2020-12-16

**Authors:** Jeffrey Stokes, Deborah Carr

## Abstract

Marriage is a dyadic system, within which the characteristics and experiences of each partner can have implications for both. Moreover, gender of both spouses may impact these dyadic influences. The five papers comprising this symposium all take a dyadic approach to studying midlife and older couples, and how their effects on one another may vary by gender. Donnelly examines the consequences of precarious work among midlife couples, finding heightened risks for marital strain and divorce, depending on which gender spouse is exposed to precarious work. Garcia also analyzes gender differences – in this case, how the gender of a woman’s spouse may affect associations between daily marital strain and sleep quality, with only women married to men showing adverse sleep outcomes. Polenick and colleagues study the long-term repercussions of chronic condition discordance, finding that both individual-level and couple-level discordance had impacts for husbands’ and wives’ physical activity. Gallagher and Stokes focus on cognitive functioning within dyads, revealing gendered effects: Wives’ poorer cognitive functioning was associated with their own (better) marital quality, while husbands’ poorer cognitive functioning was associated with wives’ (worse) marital quality. Lastly, Stokes and Barooah examine longitudinal dyadic associations between loneliness and vascular health, finding that own and partner’s baseline loneliness were associated with increased HbA1c levels only in the context of inferior marital support. Carr will assess the strengths and limitations of these papers, and discuss the contributions these studies can make to the field and to future research on marital effects and gender in later life.

